# Nadir CA-125 has prognostic value for recurrence, but not for survival in patients with ovarian cancer

**DOI:** 10.1038/s41598-021-97564-1

**Published:** 2021-09-14

**Authors:** Szymon Piatek, Grzegorz Panek, Zbigniew Lewandowski, Dominika Piatek, Przemyslaw Kosinski, Mariusz Bidzinski

**Affiliations:** 1 Department of Gynecologic Oncology, The Maria Sklodowska-Curie National Research Institute Of Oncology, Roentgen Street 5, 02-781 Warsaw, Poland; 2 1St Department of Obstetrics and Gynecology, Medical University of Warsaw, Warsaw, Poland; 3 Department of Epidemiology and Biostatistics, Medical University of Warsaw, Warsaw, Poland; 4 1St Department of Radiology, Medical University of Warsaw, Warsaw, Poland

**Keywords:** Ovarian cancer, Prognostic markers

## Abstract

The objective of this study was to evaluate the nadir CA-125 in patients with epithelial ovarian cancer. A total of 168 patients who achieved complete remission (no clinical and radiological signs, CA-125 ≤ 35 U/ml) after first-line treatment were enrolled in the study. The relationship between CA-125 and survival was examined by applying generalized additive models to the Cox proportional hazards model. The median CA-125 concentration after the treatment was 10 U/ml (2.7–35 U/ml). The nadir CA-125 was related to progression-free survival but not to overall survival. The risk of recurrence in patients with 11–25 U/ml and 26–35 U/ml compared to patients with ≤ 10 U/ml was 1.87 (*p* < 0.0024) and 2.17 (*p* < 0.018), respectively. An increased risk of recurrence according to the nadir CA-125 (≤ 10 U/ml vs. 11–25 U/ml and ≤ 10 U/ml vs. 26–35 U/ml) was found in patients with high-grade tumours (hazard ratio, HR = 2.08 and 2.59, respectively), advanced disease (HR = 2.38 and 2.03, respectively), serous histology (HR = 2.08 and 2.43, respectively) and after complete cytoreduction (HR = 2.7 and 2.72, respectively). No correlation between the CA-125 nadir and recurrence risk was found in patients with early-stage disease or those receiving neoadjuvant chemotherapy or bevacizumab.

## Introduction

The reduction of the CA-125 serum concentration to < 35 U/ml remains one of the goals of ovarian cancer (OC) treatment. The normal posttreatment level of CA-125 may be a surrogate for complete remission; however, it was found to be a poor predictor of OC regression during second-look laparotomy^1^. In advanced OC, lowering the CA-125 concentration to < 20 U/ml or even < 15 U/ml was associated with microscopic remission^2–4^. In clinical practice, measuring the serum level of CA-125 was found to be useful at different points of OC management. Increased pretreatment CA-125 levels are an independent predictor of PFS and OS^5,6^. A postoperative decline in serum CA125 levels (> 75% or ≥ 80%) was found to be an independent prognostic factor for progression-free survival^7,8^. A CA-125 decrease during neoadjuvant chemotherapy (NACT) may be used to predict cytoreductive surgery outcomes^9,10^. Finally, several authors observed longer progression-free survival (PFS) and overall survival (OS) in patients with low CA-125 nadir levels; however, different cut-off points (5–20 U/ml) were established^11–14^, and they were set arbitrarily^11,13^ according to the median^15,16^ or quartile^16^ CA125 concentration. On the other hand, a nadir of 5 U/ml is of little use in everyday practice^12^. Therefore, the clinical usefulness of the nadir CA-125 in patients with OC is not clear.

The main objective of this study was to evaluate the CA-125 nadir as a prognostic factor for survival in different subgroups of patients.

## Results

The detailed characteristics of the study group were presented in our previous paper^10^ and are shown in Table 1. Almost 70% of women were diagnosed at an advanced stage. Most cases were high grade (69.6%) and serous type tumours (74%). The median CA-125 concentration at the end of treatment was 10 U/ml (2.7–35 U/ml).

**Table 1:** Patient characteristics. The data in the table correspond to those presented in a previous study^10^.

Characteristics	Number (%)/median (range)
Age	57 (19–86)
**FIGO**	
I	42 (25%)
II	10 (6%)
III	103 (61.3%)
IV	13 (7.7%)
**Histology**	
Serous	124 (73.8%)
Endometrioid	18 (10.7%)
Clear cell	10 (6%)
Mucinous	4 (2.4%)
Nondifferentiated	4 (2.4%)
Mixed	8 (4.8%)
**Grade**	
1	16 (9.5%)
2	35 (20.8%)
3	117 (69.6%)
**Cytoreduction**	
R0	116 (69.1%)
R1 (≤ 1 cm)	29 (17.3%)
R2 (> 1 cm)	23 (13.6%)
Neoadjuvant chemotherapy	26 (15.5%)
Bevacizumab	32 (19.1%)
CA-125 before therapy	229.2 U/ml (3.8–6000 U/ml)
Nadir CA-125 after therapy	10 U/ml (2.7–35 U/ml)

**Figure 1:**
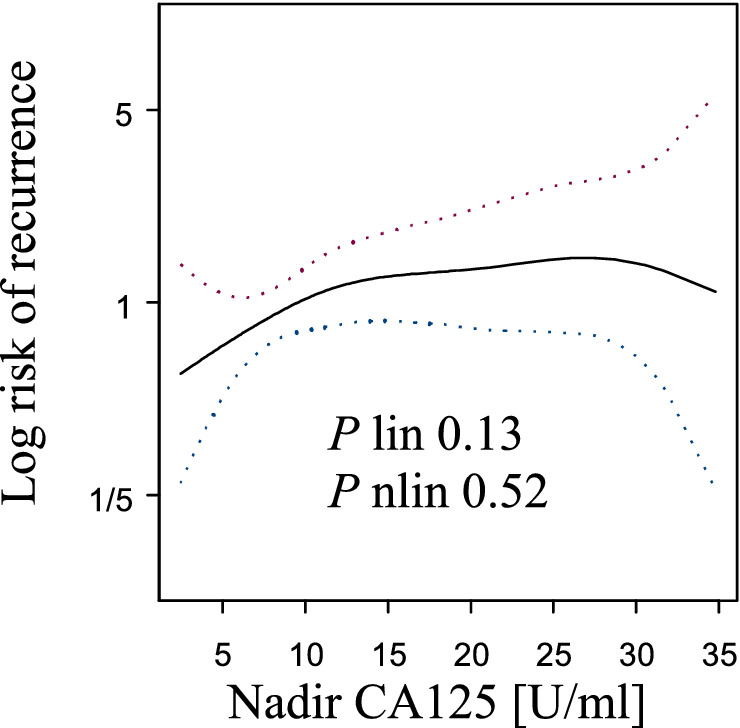
The risk of death and CA-125 nadir level were within the normal range after first-line treatment.

**Figure 2:**
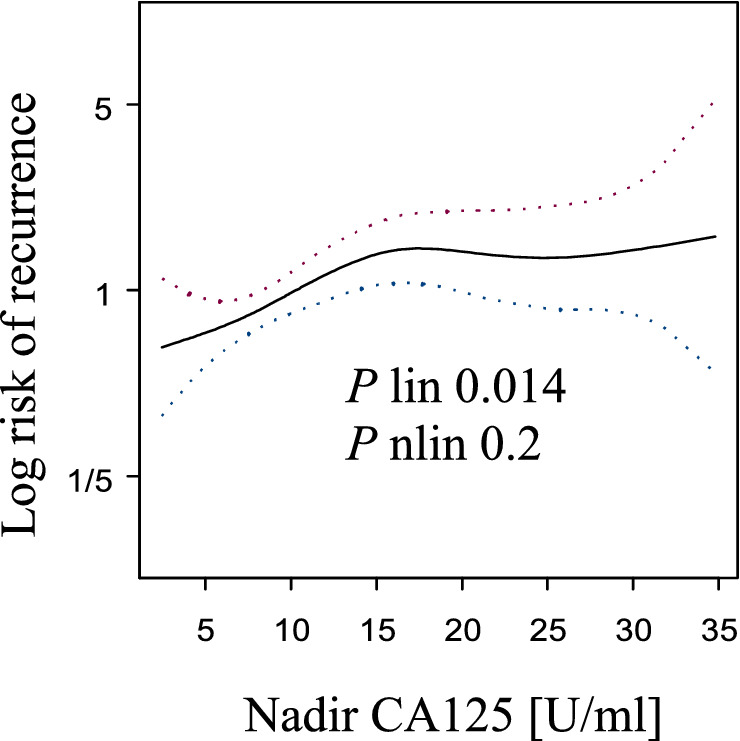
The risk of recurrence and CA-125 nadir level were within the normal range after first-line treatment. A spline indicated that the risk of recurrence initially increased, and at approximately 15 U/ml, it reached a plateau (*p* linear = 0.014).

The CA-125 nadir was not related to overall survival (OS) (*p* linear = 0.13, *p* nonlinear = 0.52; Fig. 1). A correlation between the CA-125 nadir and recurrence was found (*p* linear = 0.014, *p* nonlinear = 0.2; Fig. 2). The plot for recurrence indicated that there was a nearly linear increase in risk to ~ 15 U/ml; then, it reached a plateau with a slight increase in risk in the range of 25–35 U/ml. According to the plot, three groups of patients were identified with different CA-125 nadir levels: ≤ 15 U/ml, 16–25 U/ml, and 26–35 U/ml. In these groups, the nadir level was not related to progression-free survival (PFS) (log rank test, *p* = 0.0742).

**Figure 3:**
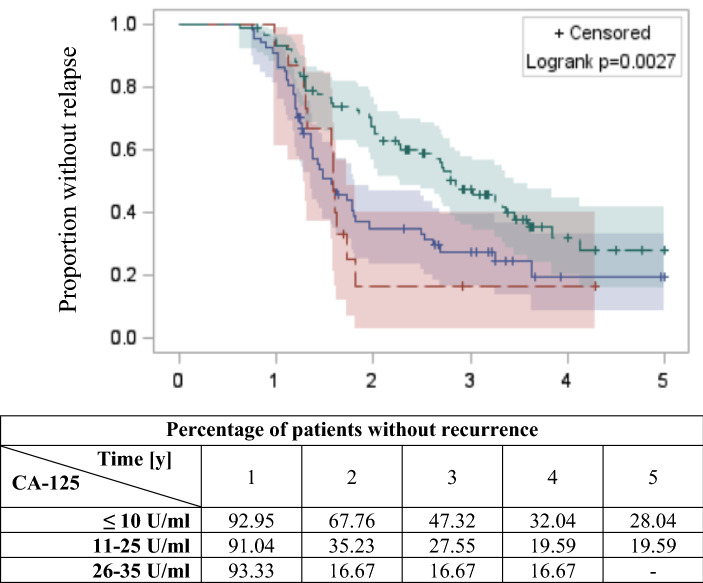
Kaplan–Meier curves of PFS for patients with different CA-125 nadir levels - green ≤ 10 U/ml (n = 86); blue 11–25 U/ml (n = 67); and red 26–35 U/ml (n = 15). A significant difference in PFS was achieved based on the log-rank test (*p* < 0.05).

Considering the median CA-125 nadir and the linear relationship between CA-125 and the risk of recurrence, the cut-off levels were changed to ≤ 10 U/ml, 11–25 U/ml, and 26–35 U/ml. Figure 3 shows the Kaplan–Meier curves for the cumulative PFS rates for patients according to the nadir level. PFS was significantly longer for patients with nadir levels ≤ 10 U/ml (Fig. 3, Table 2).

**Table 2:** Ovarian cancer recurrence risk was related to the CA-125 nadir level after first-line treatment in patients with complete remission.

CA-125 nadir	HR	95% CI min	95% CI max	*p*
≤ 10	1.00	–	–	–
11–25	1.87	1.25	2.8	< 0.0024
26–35	2.17	1.14	4.1	< 0.0178

*HR* hazard ratio, *CI* confidence interval.

### Subgroup analysis

The CA-125 nadir and recurrence risk was assessed in subgroups of patients (Table 3). The relationship between the risk of recurrence and nadir was confirmed in patients with advanced disease (FIGO III, IV), serous neoplasms, high-grade tumours (grades 2 and 3) and optimal cytoreduction. There was no correlation between the CA-125 nadir (≤ 10 U/ml vs. 11–25 U/ml and ≤ 10 U/ml vs. 26–35 U/ml) and risk of relapse in patients who started treatment with NACT (*p* = 0.49 and *p* = 0.26), received bevacizumab (*p* = 0.066 and *p* = 0.78), had locally advanced tumours (FIGO I/II; *p* = 0.75 and *p* = 0.99) or did not have elevated pretreatment CA-125 levels (*p* = 0.99).

**Table 3:** Risk of relapse and the CA-125 nadir after treatment in subgroups of patients.

Subgroup of patients	CA125 nadir after treatment		
	≤ 10	11–25	26–35
**FIGO I, II (n = 52)**			
HR	1.00	0.83	0
*P*	–	0.75	0.99
**FIGO III, IV (n = 116)**			
HR	1.00	2.38	2.03
*P*	–	< 0.001	< 0.037
**Serous histology (n = 124)**			
HR	1.00	2.08	2.43
*P*	–	< 0.002	< 0.009
**Grade 2, 3 (n = 152)**			
HR	1.00	2.08	2.59
*P*	–	< 0.001	< 0.005
**NACT (n = 26)**			
HR	1.00	1.42	2.13
*P*	–	0.49	0.26
**Bevacizumab (n = 32)**			
HR	1.00	2.23	1.2
*P*	–	0.066	0.78
**Pre-treatment CA-125 ≤ 35 U/ml (n = 13)**			
HR	1.00	0	–
*P*	–	0.99	–
**Cytoreduction (R0 and R1) (n = 145)**			
HR	1.00	2.7	2.72
*P*	–	< 0.02	< 0.05

## Discussion

In contrast to previous studies, the nadir was not related to overall survival in this study. In most of these studies, the CA-125 nadir was determined arbitrarily^11,13,17^ or based on the median^15,16,18,19^ CA-125 or quartile^16^. Riedinger et al. generated ROC curves and found that a nadir CA-125 level of 20 U/ml was predictive of PFS and OS^14^. In our study, the relationship between the nadir and risk of recurrence was investigated by applying generalized additive models to the Cox proportional hazards method. A spline illustrating the linear and nonlinear effects between the nadir and risk of death showed no relationship. Van Altena et al. observed a longer median OS in patients with a nadir ≤ 5 U/ml, but the likelihood ratio test in combination with Cox regression models showed that the nadir was an independent predictor of tumour recurrence (not for OS)^12^. The lack of a relationship between the nadir and overall survival may be due to an insufficient sample size. However, the following causes are also possible:

1. The patients in the study were more sensitive to salvage treatment due to the inclusion of fewer patients with a worse prognosis (patients with FIGO stage IV disease and who received suboptimal cytoreduction constituted only 7.7% and 13.6%, respectively).
2. The study included early-stage patients (FIGO I and II) with high survival rates (even after recurrence); a statistically significant difference was found in the survival after recurrence and the initial clinical stage (I, IIA versus IIB–IV)^20^.
3. Different patterns of recurrence were observed in analysed patients; survival after recurrence depended on the time to recurrence (≤ 6 months versus > 6 months), the number of recurrence sites (single versus multiple), and treatment at recurrence (chemotherapy plus surgery and/or radiotherapy versus chemotherapy only)^21^.
4. Thirty-two patients with advanced disease received bevacizumab, which prolonged PFS but did not impact OS.
5. The nadir CA-125 is a weak surrogate of pathologic CR, which is strongly correlated with OS; although all patients were assessed as complete responders to 1st-line treatment, it is unknown how many of them achieved a pathologic CR.

Clinicians' expectations affect the selection of patients at a particular risk of recurrence who should receive close follow-up. Van Altena found that a level of > 5 U/ml was a prognostic factor for recurrence^12^. In clinical practice, the vast majority of patients finish treatment at a level > 5 U/ml, so this level is of little clinical value for selecting high-risk patients; however, a concentration of < 5 U/ml may indicate that few patients have a low risk of recurrence. The opposite results were presented by Kang et al., who indicated that a cut-off value of 18 U/ml (90th percentile) should be used to identify high-risk patients^22^. It was found that a level of 18 U/ml had a better specificity, likelihood ratio and positive predictive value than 12 U/ml (75th percentile) and 10 U/ml (median). This finding is not surprising because they included only advanced patients (FIGO III/IV), who relapse very often. In our study, the median nadir CA-125 for patients with stage I and II disease was 9.4 U/ml, so it was almost the same as that for the whole study group. The recurrence rate in early-stage OC patients is far lower (13–30%), so the prognostic significance of the nadir CA125 is unclear in these patients. It seems that the aggressive molecular profile may be responsible for recurrence and survival in patients with an apparently good prognosis (FIGO I and II). Most previous studies examined patients with advanced OC. The present results did not find a relationship between the nadir CA-125 and risk of recurrence in early OC; however, the total number of patients with FIGO stages I and II was 52.

A linear correlation between the risk of recurrence and nadir CA-125 was found. The recurrence risk increased to ~ 15 U/ml, and then it reached plateau, with little change between 25 and 35 U/ml. Based on this spline, 15 U/ml and 25 U/ml were established as cut-off levels. However, the log rank test was not significant (*p* = 0.0742) for these borders. The presence of a relationship between the nadir and risk of recurrence suggested that new cut-off points should be identified. A value of 10 U/ml instead of 15 U/ml was chosen for several reasons. As the risk increased at 15 U/ml, it was justified to change the cut-off nadir to lower values (shift to the left side). Moreover, it reduced the disproportionate number of patients in the groups (~ 70% of patients had a nadir < 15 U/ml). The ease of application of the results in clinical practice also affected the choice of 10 U/ml as the cut-off level.

Different survival rates were observed in patients with nadirs as follows: < 10 U/ml, 11–20 U/ml and 21–35 U/ml (20–30 U/ml)^11,13^. In our study, a slightly different risk of recurrence was observed for patients with a nadir of 11–25 and 26–35 compared to a nadir < 10. These results may be caused by the limited number (n = 15) of patients with nadirs in the range of 26–35 U/ml. A direct comparison of patients with nadir levels of 11–225 U/ml vs. 26–35 U/ml showed no difference in the risk of relapse. This is consistent with the publication by Markmann et al.^13^, who found an increased recurrence risk in patients with nadirs < 10 U/ml and no differences in the risk of relapse between those with nadirs 10–20 U/ml and 20–35 U/ml.

Subgroups of patients were identified according to clinicopathological factors (Table 3). Not surprisingly, there was no correlation between the CA-125 nadir and recurrence risk in patients with normal pretreatment CA-125 levels. Although elevated levels of CA-125 are found in 50% of early-stage OC, our analysis included patients with FIGO I and II^23^. In these patients, the nadir CA-125 concentration was not helpful for predicting relapse. Another group of patients for whom the value of the nadir is unclear are patients treated with bevacizumab. In 32 patients receiving bevacizumab therapy, no correlation between the nadir CA-125 and risk of recurrence was found. Gaducci et al.^16^ included 11 patients who received bevacizumab, but they did not calculate the risk for this subgroup. Azad et al. showed that the efficacy of antiangiogenic therapy for the treatment of ovarian cancer may not correspond to the serum CA-125 level^24^. These authors concluded that caution should be exercised when using CA-125 levels in patients treated with molecular agents. Unexpected results were found in patients who received NACT because there was no correlation between the nadir CA-125 and recurrence risk. Other authors also included patients with NACT^13,17,18^; however, they did not assess the risk of recurrence in this subgroup separately. The lack of histopathological verification after treatment makes it impossible to objectively assess complete remission. Perhaps patients receiving bevacizumab and those who started treatment with NACT are less likely to achieve pathological remission, although they obtained a complete response in imaging and marker tests. This could be the result of the substantial advancement of the disease and the presence of residual disease. Our study included only 26 patients who began treatment with NACT, and these observations need to be confirmed in larger populations.

It was surprising that in patients with advanced disease, the highest risk of relapse was observed in patients with a nadir 11–25 U/ml. It was expected that the recurrence risk would increase with higher nadir levels. A possible explanation for this result is the fact that some of these patients received bevacizumab, which may have impaired the outcomes.

The strengths of our study include the following.

- All biochemical tests were performed at one certified laboratory (according to ISO 9001 Quality management systems – Requirements) with the same immunoassay (Cobas Core CA 125 II EIA assay, a one-step solid-phase enzyme immunoassay based on the sandwich principle - Roche Diagnostics). This is particularly important because a slight difference in laboratory tests may significantly affect the interpretation of the results. Previous studies were conducted at more than 1 hospital^12–14,18^ with different laboratory assays^12,14,16^.
- The analysis of consecutive patients with a complete response allowed us to assess the nadir CA-125 as an independent PFS prognostic factor regardless of stage, tumour grade, treatment (NACT, the completeness of cytoreduction, and bevacizumab) and pretreatment CA-125 levels.
- Our study included patients with any FIGO stage.

Our study had some limitations.

- A limitation of our study (and other studies on the nadir CA-125 significance) is that changes in marker concentrations may be due not only to patient improvement/disease recurrence but also to analytical imprecision and normal intra- and interindividual biological variation. Tuxen et al. found that CA-125 analytical imprecision and intra- and interindividual biological variation were 12.1%, 24.0%, and 43.1%, respectively^25^.
- Posttreatment pathologic assessments in the included patients are lacking. Complete remission was assessed clinically, biochemically and radiologically. None of these methods (separately or in combination) correspond to histological remission.
- As a retrospective analysis, selection bias may have occurred. Some subgroups of patients contained a limited number of patients.

CA-125 is a well-known biomarker, but its significance is still of interest to clinicians and researchers. Recently, the role of nadir CA-125 and its glycoforms have been assessed by Finnish authors^26^; the results of their study may undermine the prognostic significance of nadir CA-125^26^. The impact of new therapies (PARP inhibitors, immunotherapy) on clinical significance of nadir CA-125 is unknown. As the management of ovarian cancer changes, assessment of the CA125 serum concentration during treatment and follow-up should be explore.

## Conclusions

The nadir CA-125 had prognostic value for recurrence in patients with advanced FIGO stages who underwent primary cytoreductive surgery followed by chemotherapy and achieved a complete response.

If disease advancement requires treatment with NACT and/or bevacizumab, the prognostic significance of the nadir CA-125 may be unclear.

Recurrence and survival in patients with early-stage disease are not related to the nadir CA-125. The prognosis of these patients depends on the unfavourable molecular characteristics of the tumour rather than biochemical results.

## Material and methods

The study group was described in detail in a previous paper^27^. Briefly, 168 patients with epithelial ovarian cancer who achieved a complete response after first-line treatment were enrolled in the study. All patients underwent surgery and chemotherapy based on a combination of carbo-/cisplatin with paclitaxel. Complete response was confirmed clinically (no signs and symptoms of disease), radiologically (RECIST) and biochemically (CA-125 < 35 U/ml). The recurrence of the disease was diagnosed at the first appearance of symptoms: clinical, radiological or histopathological/cytological.

The nadir CA-125 concentration was measured in serum within one month after treatment. CA125 was measured with an electrochemiluminescence immunoassay (Roche Diagnostics). All biochemical tests were conducted at one laboratory.

The Bioethical Committee of the Medical University of Warsaw approved this study (No AKBE/26/2018). All methods were performed in accordance with the relevant guidelines and regulations. Patients provided informed consent for the use of medical data in the study.

### Statistical analyses

The data are expressed as percentages, means and medians. The percentage of women without recurrence during the 5-year observation period is presented with the Kaplan–Meier (K-M) method and by plotting relevant curves. The relationship between CA-125 and the risk of recurrence was examined by applying generalized additive models to the Cox proportional hazards model to generate curves illustrating both the linear and nonlinear relationship of the examined parameter with the estimated recurrence risk. The emergence of a statistically significant nonlinear effect was the basis for an approximate determination of the limit values of CA125, which in turn were used to identify subgroups of patients for whom K-M curves were drawn. The methodological aspects of the study were based on the textbook by van Belle et al.^28^, while SAS/STAT® 9.4/14.4, User's Guide, SAS Institute Inc., Cary, NC, USA, 2017 was used to address the technical and methodological aspects.
